# Physical activity in older age: perspectives for healthy ageing and frailty

**DOI:** 10.1007/s10522-016-9641-0

**Published:** 2016-03-02

**Authors:** Jamie S. McPhee, David P. French, Dean Jackson, James Nazroo, Neil Pendleton, Hans Degens

**Affiliations:** School of Healthcare Science, Manchester Metropolitan University, John Dalton Building, Manchester, M1 5GD UK; School of Psychological Sciences, University of Manchester, Manchester, UK; Faculty of Life Sciences, University of Manchester, Michael Smith Building, Manchester, UK; School of Social Sciences, Humanities, University of Manchester, Manchester, UK; Clinical Gerontology, Salford Royal Hospital & University of Manchester, Manchester, UK; Lithuanian Sports University, Kaunas, Lithuania

**Keywords:** Exercise, Physical activity, Muscle, Falls, Health, Frailty

## Abstract

Regular physical activity helps to improve physical and mental functions as well as reverse some effects of chronic disease to keep older people mobile and independent. Despite the highly publicised benefits of physical activity, the overwhelming majority of older people in the United Kingdom do not meet the minimum physical activity levels needed to maintain health. The sedentary lifestyles that predominate in older age results in premature onset of ill health, disease and frailty. Local authorities have a responsibility to promote physical activity amongst older people, but knowing how to stimulate regular activity at the population-level is challenging. The physiological rationale for physical activity, risks of adverse events, societal and psychological factors are discussed with a view to inform public health initiatives for the relatively healthy older person as well as those with physical frailty. The evidence shows that regular physical activity is safe for healthy and for frail older people and the risks of developing major cardiovascular and metabolic diseases, obesity, falls, cognitive impairments, osteoporosis and muscular weakness are decreased by regularly completing activities ranging from low intensity walking through to more vigorous sports and resistance exercises. Yet, participation in physical activities remains low amongst older adults, particularly those living in less affluent areas. Older people may be encouraged to increase their activities if influenced by clinicians, family or friends, keeping costs low and enjoyment high, facilitating group-based activities and raising self-efficacy for exercise.

## Background

**Fig. 1:**
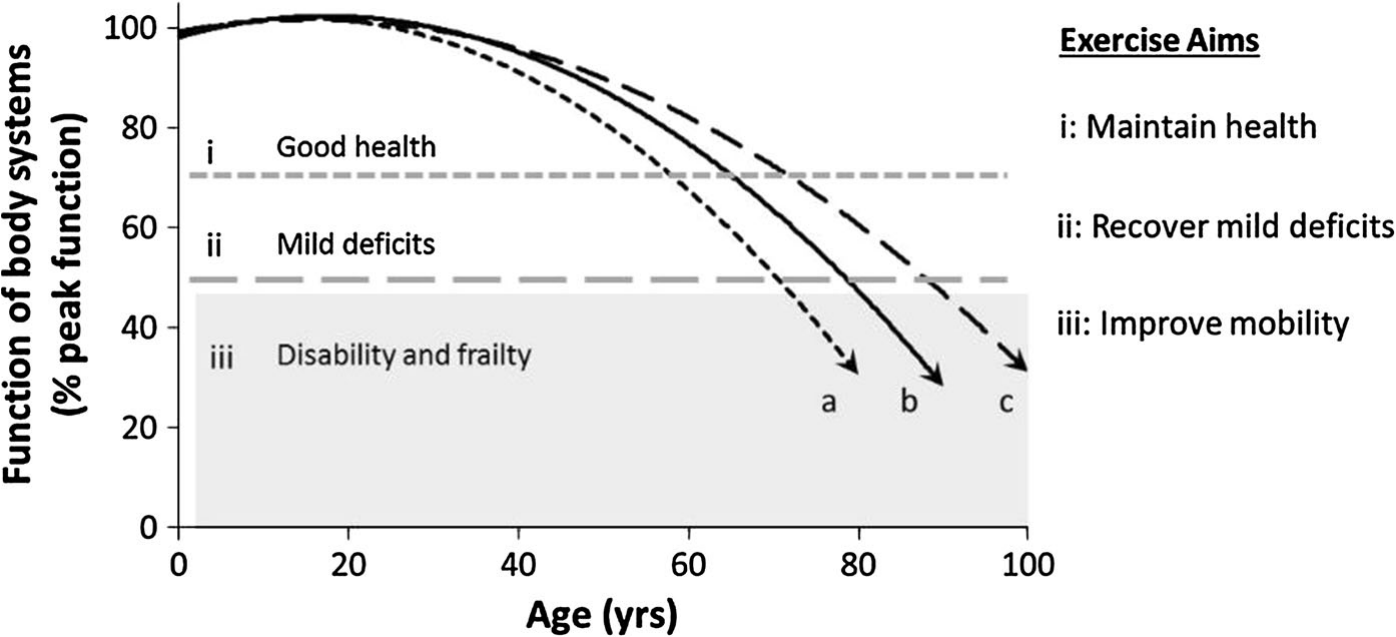
Schematic representation of ageing trajectories and individual exercise needs. Good physiological function is maintained until middle age and thereafter progressively deteriorates. The *upper horizontal dashed line* represents a theoretical point at which deterioration manifests as moderate functional deficits and above this line the general aim of physical activity is to maintain good health. The *lower horizontal dashed line* indicates a theoretical threshold beyond which a person suffers disability and frailty, so the aim of physical activity is to recover the deficits and improve mobility. The *curved lines* represent *a* accelerated ageing, *b* normal ageing and *c* healthy ageing. Exercise interventions should match the physical capability, rather than chronological age *per se* to be effective

Data from the UK Office for National Statistics (2012) project an increase in the population aged over 60 years from 17 % in 2010 to around 23 % by 2035. The most rapid rise is projected for the ‘oldest’ old, where the number of people aged over 85 years increases from 1.4 million to around 3.5 million. A general schematic representation of ageing is shown in Fig. 1. After the age of around 40 years it is possible to detect deterioration of the function of physiological systems, with associated anatomical and ultrastructural changes. For instance, progressive cognitive declines affect memory and learning; skeletal muscle atrophies and becomes progressively weaker (known as sarcopenia) and ageing-related declines in bone mineral density lead to osteopenia and osteoporosis. Chronological age is a convenient and often very good predictor of health status, disease burden and physical capability, but there is considerable inter-individual variability, with some older people having very good health and others show accelerated onset of weakness, disability and frailty.

The lifestyle and medical advances that contribute to longevity are achievements to celebrate, but they also bring unintended and considerable social, economic and health challenges as life expectancy increases faster than the period of life spent in good health, termed ‘healthy life years’ (discussed in (Rechel et al. 2013)). For instance, in the UK the mean life expectancy for women at birth in 2008 was 81.8 and in men it was 77.7 years and by 2013 this had increased to 82.9 for women and 79.2 years for men. Over this same time period, the mean healthy life expectancy had changed little, or even decreased: for women in 2008 it was 66.3 and in men it was 65 years, but by 2013 it had fallen to 64.8 in women and 64.4 years in men (Eurostat 2015). Musculoskeletal disorders are the most common chronic, disabling conditions, affecting 14 % of people aged over 65 years. These are followed by heart and circulatory conditions affecting 10 %; respiratory conditions affecting 6 %; endocrine or metabolic conditions affecting 6 % and mental disorders affecting 4 % of people aged over 65 years. Incidence of these chronic diseases more than doubles in the 10 years that follow retirement. Of people aged over 75 years, 30 % report chronic musculoskeletal conditions; 32 % report heart and circulatory conditions and 13 % report endocrine or metabolic conditions (ONS 2013). Another of the concerns is that the ‘old age dependency ratio’ will change from four working-age persons for every one older retired person, to just two working-age persons for every one older retired person in Europe (Eurostat 2014). This change in the ratio of workforce to the overall population may strain economic, social and healthcare support systems, so it is important to implement strategies to improve the health of older people.

## Healthy ageing and frailty

*Healthy ageing* has been defined as an ability to lead a healthy, socially inclusive lifestyle relatively free from illness or disability (age UK 2010), and this is more likely in those actively engaging in activities to improve their health and wellbeing (age UK 2015). Such people were recruited into the MYOAGE study (McPhee et al. 2013) and it was clear that despite no obvious difficulties with usual activities of daily living, the vast majority of older people aged in their 70 s had lower physiological function than young adults. For example, older adults had higher body mass index due to increased fatness, smaller and weaker muscles particularly in the legs, lower bone mineral density, reduced cardiorespiratory and metabolic function and performed worse in cognitive tests than young (Bijlsma et al. 2013a, b; Sillanpaa et al. 2014). Other studies have shown 30–50 % fewer motor neurons innervating leg muscles of healthy old compared with young, suggesting that motor unit remodelling is a part of the normal ageing process (Campbell et al. 1973; Piasecki et al. 2015a, b). The motor neuron and muscle fibre (Lexell et al. 1988) losses occurring during ageing can never be replaced, but the structure and function of cardiorespiratory, metabolic and musculoskeletal systems are amenable to improvement through exercise training, so it is informative to profile very athletic older people (master athletes). Master Athletes regularly compete in sports and demonstrate exceptional physical capability for their age (Rittweger et al. 2009). They usually retain greater bone, muscle, cardiorespiratory, metabolic and neuronal health compared with non-athletic people of similar age, but it is nevertheless evident that physiological systems decline in older age even in those who remain exceptionally active (Degens et al. 2013a, b; Ireland et al. 2014; Michaelis et al. 2008; Pearson et al. 2002; Power et al. 2010; Trappe et al. 2013; Wilks et al. 2009).

The progressive declines in physiological function that usually occur over decades are associated with slower walking speed and difficulties rising from a seated position and balancing. Standardised assessments have been developed to indicate physical capability in older age, including the 6-min walk (Rikli and Jones 1998) and the 30 s chair-rise test (Jones et al. 1999). The Short Physical Performance Battery assesses normal walking speed over 4 m, balancing in different foot positions and time to complete 5 chair-rises, and the maximum score of 12 is easily achieved by healthy older people, while a score less than 8 indicates sarcopenia and frailty (Pahor et al. 2006). Thus, a score of between 8 and 11 indicates the moderate physical impairments associated with sarcopenia (low muscle mass) and ‘pre-frailty’ (Bauer 2015). The Timed Up and Go (TUG) test involves standing from a seated position, walking around a cone placed 3 metres away and returning to a seated position on the original chair. Healthy men and women complete the task within 7 s (Bijlsma et al. 2013a) and frail people take >10 s (British Geriatric Society 2014), suggesting that a score between 7–10 s is indicative of pre-frailty.

Frailty is recognised clinically as a geriatric syndrome that arises due to multiple deficits to body systems. Frail people experience severe impairments to physical and mental function that restrict their ability to complete necessary activities of daily living. Frailty is usually diagnosed according to two classifications. The *Rockwood scale* describes frailty as an accumulation of ‘deficits’, including the number of medications taken, number of diseases, frequency of medical interventions and other psychosocial indicators (Rockwood et al. 2005). The *Fried Frailty Phenotype* recognises frailty in people presenting at least three of the following five conditions: unintentional weight loss; low physical activity levels; slow gait speed; exhaustion, and weakness (Fried et al. 2001). Around 10 % of people aged 65–75 years and half of all people aged over 80 years suffer from frailty, which is aggravated by a lower social status, comorbidities, medication use and lowered immunity (Ashfield et al. 2010; Clegg et al. 2013; Syddall et al. 2010; Yao et al. 2011). There is a wide spectrum of frailty, but typically, frail people have low physical activity, few social interactions as well as several chronic diseases that require medical attention (Marengoni et al. 2011). They are vulnerable to falling and may not fully recover from mild stressors or illness. Frailty can be a dynamic state, as some people with high levels of dependency or disability can recover independence, although they remain at higher risk of future mobility limitations than those who were never frail (Gill et al. 2006; Hardy and Gill 2004). Some recommended indicators of frailty are shown in Table 1, which are possible predictors of future falls (Ganz et al. 2007).

**Table 1:** Indicators of physical frailty and their measurement

Indicator	Measurement	Reference
Walking	<0.8 m/s or taking more than 5 s to walk 4 m	BGS (2014)
	Inability to walk half-a-mile or negotiate stairs	Dufour et al. (2013)
Standing	>10 s in the ‘timed up and go’ test	BGS (2014)
	>30 s to complete 5 × chair rise	
Muscle strength and power	Men grip strength: <37 kg	Sallinen et al. (2010)
	Women grip strength: <21 kg	
	Standing jump <8 cm	Runge et al. (2004)
Balance	<10 s standing on one leg	
Activities of daily living	Difficulties to complete heavy housework	Dufour et al. (2013)
	Sedentary lifestyle and social isolation	Fried et al. (2001)
	Poor coordination of movements	Daniels et al. (2008)
Self-reported health	Scoring >3 on the PRISMA 7 questionnaire	BGS (2014)
	>3 kg unintentional weightloss in last 3 months	
	Chronic exhaustion or fatigue	Fried et al. 2001

Cut-off values indicate the level of physical functioning and health status

## Regular physical activity to promote healthy ageing

In general, the more often a person is physically active, the better their physical capability. This is due to adaptations of physiological systems, most notably within the neuromuscular system to coordinate movements, the cardiopulmonary system to more effectively distribute oxygen and nutrients around the body, and metabolic processes particularly those regulating glucose and fatty acid metabolism, which collectively increase overall aerobic power and physical capability. Thus, the trajectory towards frailty is directly modifiable through physical activity habits (Department of Health 2011; Health 2009; Tak et al. 2013).

**Fig. 2:**
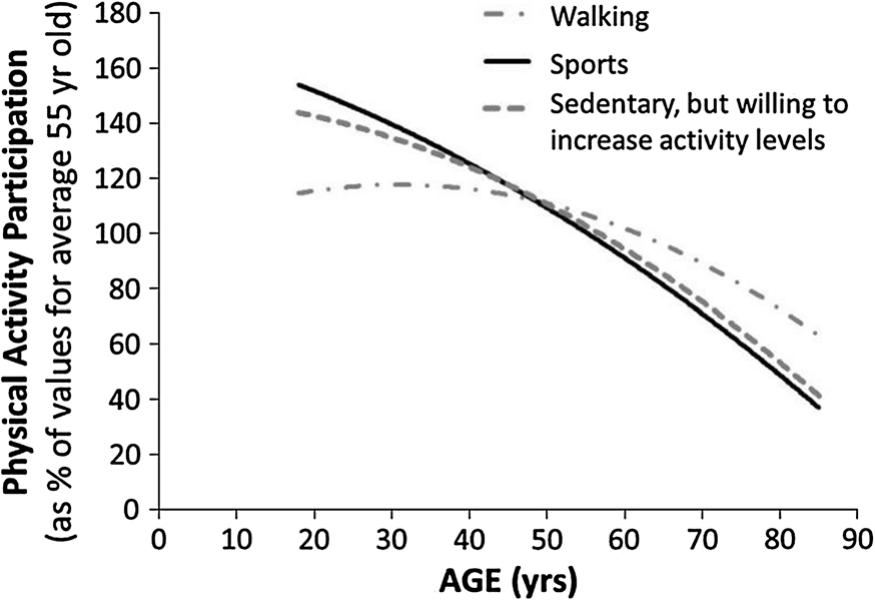
Physical Activity Participation in UK adults. With increasing age, sports participation progressively declines. Walking for health benefits or enjoyment remains fairly constant amongst young and middle-aged adults, but declines progressively into older age. Amongst those who are sedentary, more of the younger adults have a desire to increase physical activity levels compared with the middle-aged and the old. Data are from The Taking Part Survey, which interviewed >92,000 people in England between 2005 and 2009 to ask about physical activity habits (Department for Culture 2011)

A survey of >92,000 people in England showed that exercise participation declines progressively throughout adult life and so does the desire to participate (Department for Culture 2011). Indeed, only around half of all adults and just a quarter of people aged over 65 years meet the *minimum* recommended activity levels needed to maintain health (Department of Health 2011). Inactivity is the major cause of poor physiological fitness and disease in older age, at least equal to the effects of smoking, drinking excessive alcohol intake and obesity (Booth et al. 2000; Lee et al. 2012). Sedentary people aged 50 years and older had twice the risk of death compared with those who had the highest level of physical activity after adjusting for a range of risk factors (including age and socio-economic position) (Nazroo et al. 2008). For example, those who retire from work are more likely than those who remain in work to change to low levels of physical activity from both high and medium levels (Matthews et al. 2014) and people aged 70–79 years are about half as likely as those aged 50 to 59 years to be engaged in high levels of physical activity (Matthews et al. 2014). People aged >80 years are over 50 % less likely than those in their early 50 s to engage in sports or to want to increase their activity levels (Fig. 2).

People with higher activity levels and physiological fitness have a lower mortality risk (Feldman et al. 2015). Maintenance of a physically active lifestyle through middle and older age is associated with better health in old age (Hamer et al. 2014) and longevity (Manini et al. 2006; Stessman et al. 2009). Beginning a new exercise regimen in middle age is associated with healthy ageing (Sabia et al. 2012; Sun et al. 2010). But, even for those who were relatively sedentary through middle age, it is never too late because beginning a new exercise regimen in old age leads to significant improvements to health (Berk et al. 2006; Hamer et al. 2014) and cognition (Lautenschlager et al. 2008). Physical activity reduces the risk of developing cardiovascular and metabolic disease through better control of blood pressure, cholesterol and waist circumference in a dose-dependent manner: more activity leads to lower risk of cardiovascular and metabolic disease (Earnest et al. 2013). The metabolic benefits of increasing fatty acid oxidation in skeletal muscle, rather than accumulating intramuscular and adipose tissue stores around the major organs as well as lowered blood pressure helps to reduce the risk of developing type 2 diabetes mellitus and cardiovascular disease (Roberts et al. 2013; Stewart et al. 2005). In the nervous system, regular exercise helps to maintain cognitive function (Lautenschlager et al. 2008) and possibly also the numbers of peripheral motor neurons controlling leg muscles (Power et al. 2010, 2012) and overall improves balance and coordination to reduce risk of falls (Franco et al. 2014; Gillespie et al. 2012; Rubenstein et al. 2000). Should a fall occur, people who exercise regularly (particularly weight bearing activities that include higher impacts) are less likely to suffer a bone fracture because their bones are stronger and have higher bone mineral density (Ireland et al. 2014).

## The general aims of physical activity programmes

The National Institute for Health and Care Excellence recommend exercise as primary care (NICE 2009), but knowing how to encourage exercise participation at the population-level is challenging because a one-size-fits-all programme is not suitable. The intensity of exercise should be modified to appropriately match the individual’s exercise experience and physical capability, as indicated in Fig. 1. To be most effective, it is important that exercise programmes are appropriately designed and focus on a range of outcomes, not simply weight loss, as improved health and mobility in exercising older people can occur independently of changes to body mass index (Bruce et al. 2008). Achieving >150 min/week moderate-intensity aerobic exercise, such as walking or other moderate intensity aerobics-type activities, is associated with at least 30 % lower risk of morbidity, mortality and functional dependence compared with being inactive (Chou et al. 2014; Paterson and Warburton 2010). Walking 5–7 days per week was associated with 50–80 % lower risk of mobility impairments (Clark 1996; Roh and Park 2013) and increases longevity by around 4 years and disability-free life expectancy by around two years (Ferrucci et al. 1999). There is also evidence that sedentary people will benefit from regular short activity periods of as little as 1 min (Healy et al. 2008) or 10 min bouts (Powell et al. 2011) to break-up periods of sitting or lying.

Although vigorous activities are not advisable for sedentary older people, masters athletes can train and compete in very high intensity sports, with the risks of adverse events during competition being similar to those of younger adults (Ganse et al. 2014). Older people who maintained regular jogging postponed disability by almost 9 years and had three times lower risk of death compared to those who had never been a member of a running club (Wang et al. 2002). The risk of developing cardiovascular disease is also lower in those completing regular vigorous compared with moderate intensity exercise (Swain and Franklin 2006). Thus, there appears to be a dose–response relationship to indicate that higher intensity activities bring greater health benefits (Bruce et al. 2008; Ebrahim et al. 2000; Kim et al. 2013; Wannamethee et al. 2005), but it is important to note that older people must be suitably adapted to participate in the higher intensity activities, so this relationship might reflect the overall exercise history.

It is advisable for older people to perform activities aimed at increasing the size and strength of their limb muscles in order to combat the effects of sarcopenia, the loss of muscle mass with ageing (Maden-Wilkinson et al. 2013; Rosenberg 1997). Moderate and high intensity strength training (using a resistance of between 60–80 % of the maximal strength) increase muscle size, strength and power, even in very elderly and frail people (Fiatarone et al. 1990; Harridge et al. 1999). This is important since low muscle mass and power are associated with mobility impairments in older age (Dufour et al. 2013; Maden-Wilkinson et al. 2014). There is a dose–response relationship, meaning that higher intensity activities tend to lead to greater gains in muscle mass, strength and power than lower intensity activities (Steib et al. 2010). An expectation may be that gains in strength and power will improve walking, chair rising and stair negotiation, but several studies failed to confirm this (Beijersbergen et al. 2013; Paterson and Warburton 2010; Steib et al. 2010). However, the majority of resistance training studies were designed to target the muscles of the thigh and upper body, so the common tests of mobility might not be sensitive to show the effects on overall mobility. It is also important to train the ankle plantar flexors (calf muscles) since loss of power in this muscle group is associated with slower walking speed (Beijersbergen et al. 2013; Stenroth et al. 2015), and increased power with training improves balance (Orr et al. 2006) and mobility (Pereira et al. 2012).

Activities for frail older people should be adapted accordingly. Reviews of the literature (Forster et al. 2010; Weening-Dijksterhuis et al. 2011) led to the recommendations that frail older people should perform moderate-intensity leg-strengthening exercises and functional training, including walking, chair rising, balancing and game-like activities, two to three times per week with sessions lasting around 45 min. This is in line with the suggestion that combined resistance and endurance training may be more beneficial than any of these exercise types individually for improving functional mobility, walking, balance, reducing falls risk and risk of developing metabolic and cardiovascular disease among older people with moderate deficits or frailty (Buchner et al. 1997; Davidson et al. 2009). The combination of strength and endurance training improved muscle, cardiorespiratory and metabolic health which all contributed to improved quality of life (Chin et al. 2004, 2006; Holviala et al. 2012; Sillanpaa et al. 2008, 2012). In men and women aged 70–89 years who were sedentary, but with moderate deficits and at high risk of disability, a 12-month combined training programme improved mobility significantly more than a healthy ageing educational programme (Pahor et al. 2006). Furthermore, in frail older people, 12 month combined training (aerobic, strength, balance and flexibility) was more effective than conventional aerobic training alone at improving general activity levels (Molino-Lova et al. 2013) and functional mobility (Fielding et al. 2007; Pahor et al. 2014), and reduced risk of mobility disability by around 30 % (Pahor et al. 2014).

## Risks and adverse events

The UK Department of Health (2011), US Department of Health (2009, 2011), American College of Sports Medicine (1998), World Health Organisation (2010) and European Association of Cardiovascular Prevention and Rehabilitation (Borjesson et al. 2011) guidelines state that exercise is generally safe for older people and they therefore need not consult a medical practitioner before increasing physical activity levels. Nevertheless, as cardiovascular risks, such as increased blood pressure, arrhythmia or myocardial infarction are concerns when taking up exercise, the European Association of Cardiovascular Prevention and Rehabilitation suggest self-assessment by a brief questionnaire (Borjesson et al. 2011) to determine the need for advice from health professionals. In most cases, this is precautionary and a medical practitioner will allow the person to proceed with moderate exercise. Training intensity or exercise duration should be increased modestly not more than once every 4 weeks (Huang et al. 2005).

Exercise classes to improve balance are not associated with increased risk of adverse events. However, more intense falls-prevention classes may have an increased risk of muscle soreness or swollen joints in sedentary people unaccustomed to exercise (Gillespie et al. 2012; Howe et al. 2007). Frail or sedentary older adults living in care may have a small increased risk of falls shortly after falls-prevention classes, possibly related to physical or mental fatigue, but there is no evidence of serious adverse outcomes, injury or cardiovascular events (Crocker et al. 2013). Exercise interventions to improve balance in those diagnosed with dementia bring numerous benefits without an increased risk of adverse outcomes (Forbes et al. 2013).

Risks associated with resistance training have been reviewed in two reports (Liu and Latham 2009, 2010). The vast majority of clinical trials did not report any adverse events after exercise. It is not possible to know whether this was because no adverse events occurred or whether they were not reported. In trials that did report adverse events, the most common were minor musculoskeletal problems such as pain in joints, bruising or sprains. Less common were cardiovascular events, with only one occurrence out of the 58 reviewed trials that included people aged >60 years that did report any adverse events (Liu and Latham 2010). There was a higher risk of any adverse event in older people after intense exercise in those who already experienced pre-existing health problems, were functionally limited or were sedentary (Liu and Latham 2010).

Low and moderate intensity aerobic exercise are low risk for older people and even more intense aerobic activities carry relatively little risk. Several studies reported no greater risk of adverse events from moderate exercise compared with those not participating in physical activities (Church et al. 2007; Dunn et al. 1999; King et al. 2002). In a study of 1635 older people with moderate mobility impairments, a large-scale mixed aerobic, resistance and balance exercise intervention reported a 8 % higher incidence of serious adverse events compared with a (sedentary) health education programme (Pahor et al. 2014). An aerobic exercise intervention aiming for 60 min intense cycling/walking/running/rowing exercise 6 days per week over 12 months for previously sedentary men aged 40–75 years found no increased risk of injury or adverse event. In the non-exercise control group, 27 % experienced an injury compared with 28 % in the exercise group (Campbell et al. 2012).

Cardiovascular events during intense exercise have been estimated to occur at a rate of around 1 event per 100 years of vigorous activity (Powell et al. 2011). Risks tend to be highest during the first few weeks of a new vigorous training programme (Mann et al. 1969). However, for older people well-accustomed to intense exercise, participating in competitive vigorous sports does not carry higher risk compared with those faced by younger adults (Ganse et al. 2014). Extreme endurance running, such as a marathon, carried just 0.0005 % risk of sudden cardiac arrest across the population of runners, including older runners, equivalent to around five incidents per one-million runners (Kim et al. 2012). Because exercise has many positive effects, the overall risk of adverse events (covering all activities in the day) was approximately halved in people who achieved >140 h vigorous activity per week compared with sedentary people (Siscovick et al. 1984), demonstrating a clear net decrease in adverse events in healthy and active people compared with sedentary.

## Social, demographic and psychological considerations

Exercise habits differ depending on income, gender, age, ethnicity and disability (Department of Health 2011). Older people in higher socioeconomic positions are more likely to maintain high levels of physical activity. Those in lower socioeconomic positions are more likely to remain inactive, to move from high levels of physical activity to low levels of physical activity, and to move from medium levels of physical activity to low levels (Matthews et al. 2014). These data support others showing clear social and demographic influences on exercise habits (Evans and Kantrowitz 2002; Evans and Kim 2010; Menec et al. 2010; Salas 2002). The progression towards physical disability and frailty increases after retirement (Iparraguirre 2014; Stenholm et al. 2014b) and evidence from the United States and Europe suggests that poverty (Wahrendorf et al. 2013) and underlying disease increase the risk of physical disability in a dose–response manner (Stenholm et al. 2014a). People from more affluent backgrounds are almost three times more likely to be healthy in older age (Hamer et al. 2014) compared with those from poorer communities and a strong relationship exists between socio-economic position and health in older age (physical, psychological and overall frailty) (Banks et al. 2006; Marmot et al. 2003). Although the strength of this relationship reduces with age, this appears to largely be a consequence of higher mortality rates amongst the most vulnerable in lower socioeconomic groups (McMunn et al. 2009). Indeed, longitudinal studies examining the onset of illness and/or mortality among older people who were initially healthy shows marked increases in risk with decrease in socioeconomic position (McMunn et al. 2009).

In addition to the social and demographic associations with healthy behaviours, psychological factors are also important. The internal motivations for sports participation amongst older people include the health, social, mental and emotional benefits that help to maintain physical independence (Sport-England 2006). External motivation comes from the media, doctors, partners, friends and/or family. Provision of local opportunities and an exercise ‘buddy’ also help. The most common barriers to exercise are costs, lack of time, and physical limitations. Other limiting factors included cultural ‘norms’, language barriers and the need for clothing that may be deemed inappropriate (Sport-England 2006). Older people felt that the best way to increase participation would be to keep costs low, make sessions enjoyable, be reassured about the safety of activities and the opportunities to be physically active could be better advertised (raise awareness of local exercise classes) (Sport-England 2006).

Other less modifiable individual factors can predict initiation and maintenance of physical activity. For instance, a better physical and mental health, cognitive functioning, lower age, and higher baseline physical activity are associated with maintenance of physical activities (Koeneman et al. 2011; van Stralen et al. 2010). Of the individual factors investigated, the most consistent predictor of physical activity (this differs from sports participation) initiation and long-term maintenance is self-efficacy (French 2013; Koeneman et al. 2011; van Stralen et al. 2010). Self-efficacy for physical activity can be thought of as the belief in one’s capabilities to organize and execute that behaviour, or the belief that performing physical activity is under one’s control and may be easy (Bandura 1997). It is possible to increase people’s self-efficacy by asking them to successfully perform a behaviour in a safe environment, recalling successfully performing it before, or seeing others perform the behaviour (Darker et al. 2010; French et al. 2014). Another factor that is important in the initiation of physical activity is a person’s expectations that the activities will result in positive outcomes (van Stralen et al. 2010). These expectations may relate to health, social or other desired outcomes. People who have more social goals may choose activities such as group walks, whereas those who are concerned about falling may choose more structured programmes that directly address balance. Importantly, where people are satisfied with the outcomes they originally desired, they are more likely to continue regular physical activity (Kassavou et al. 2014).

As people get older, they are less interested in improving their health, but more interested in retaining the health and capacities they already possess (Lockenhoff and Carstensen 2004). Given this, it is important for physical activity programmes to reassure potential participants that they are unlikely to incur injuries or otherwise harm themselves. Equally, rather than promoting physical activity programmes on the basis of health improvements, many people will be more interested in activities that they view as being intrinsically enjoyable, such as interactions with other people who are also performing the activities (Devereux-Fitzgerald et al. 2016). These group activities are likely to be satisfying and become habitual through repetition (Gardner 2013). Women tend to engage more with walking groups (Kassavou et al. 2013), while men may tend to value sports, especially if it relates to teams they support (Hunt et al. 2014). As well as these individual factors, participation in physical activity is more likely when significant others approve, when people have larger social networks and when social norms amongst their peers includes being physically active (Koeneman et al. 2011; van Stralen et al. 2010). Similarly, at the wider societal level, more people are likely to be physical activity when costs are low and a wide variety of physical activity opportunities are available (Sallis and Owen 1998). Finally, physical activity can be stimulated by features of the built environment, such as safe foot- or cycle-paths and parks, and societal norms and practices that contribute to increased physical activity (Sallis and Owen 1998).

## Conclusions

The evidence shows that regular physical activity is safe for healthy and for frail older people and the risks of developing major cardiovascular and metabolic diseases, obesity, falls, cognitive impairments, osteoporosis and muscular weakness are decreased by regularly completing activities ranging from low intensity walking through to more vigorous sports and resistance exercises. Yet, participation in physical activities remains low amongst older adults, particularly those living in less affluent areas. Older people may be encouraged to increase their activities if influenced by clinicians, family or friends, keeping costs low and enjoyment high, facilitating group-based activities and raising self-efficacy for exercise.
